# Muter’s Analytical Chemistry

**Published:** 1891-11

**Authors:** 


### Book Reviews.

MUTER'S ANALYTICAL CHEMISTRY. By John Muter, M. A., Ph. D., F. R. S. E., etc.: Published by P. Blakis ton, Son & Co., Philadelphia, Pa.

This work after passing through four English editions, comes to us in its first American edition, edited by Claude C. Hamilton, M. D., Ph, G.

As stated in the preface, the editor has altered the original text as little as possible. As its name implies, the book is intended for the laboratory only. After devoting a chapter to the explanation of the more ordinary processes employed by the analyst, the author takes up the detection of the metals, arranging them in groups and giving the wet and dry reactions for each metal. He then gives the methods for the detection and separation of acidulous radicals, of both the mineral and organic acids. The fourth chapter is devoted to qualitative analysis as applied to the detection of unknown salts, with full tables for the separation of the metals of different groups. Next follows the detection of the alkaloids and their salts used in medicine. The author then enters quantitative analysis, both volumetric and gravunetric. Devotes a chapter to the analysis of water, air and food, also a chapter to the analysis of drugs, and closes with a chapter upon the analysis of urine. The work is one to be commended to the laboratory student and we think will admirably meet the wants of both teacher and pupil. The book is well printed upon good paper, fully indexed and of a size to be easily handled. We trust that it will find its way to the desk of all students engaged in analytical work. L. H. J.

